# Enabling reproductive, maternal, neonatal and child health interventions: Time trends and driving factors of health expenditure in the successful story of Peru

**DOI:** 10.1371/journal.pone.0206455

**Published:** 2018-10-31

**Authors:** Luis Huicho, Carlos A. Huayanay-Espinoza, Patricia Hernandez, Jessica Niño de Guzman, Maria Rivera-Ch

**Affiliations:** 1 Centro de Investigación para el Desarrollo Integral y Sostenible, Universidad Peruana Cayetano Heredia, Lima, Peru; 2 Centro de Investigación en Salud Materna e Infantil, Universidad Peruana Cayetano Heredia, Lima, Peru; 3 School of Medicine, Universidad Peruana Cayetano Heredia and Universidad Nacional Mayor de San Marcos, Lima, Peru; 4 Netherlands Interdisciplinary Demographic Institute, Rotterdam, The Netherlands; 5 Ministry of Economy and Finance, Lima, Peru; University of Waterloo, CANADA

## Abstract

We compared expenditure trends for reproductive, maternal, neonatal and child health (RMNCH) with trends in RMNCH service coverage in Peru. We used National Health Accounts data to report on total health expenditure by source; the Countdown database for trends in external funding to RMNCH, and Ministry of Finance data for trends in domestic funding to RMNCH. We undertook over 170 interviews and group discussions to explore factors explaining expenditure trends. We describe trends in total health expenditure and RMNCH expenditure in constant 2012 US$ between 1995 and 2012. We estimated expenditure to coverage ratios. There was a substantial increase in domestic health expenditure over the period. However, domestic health expenditure as share of total government spending and GDP remained stable. Out-of-pocket health spending (OOPS) as a share of total health expenditure remained above 35%, and increased in real terms. Expenditure on reproductive health per woman of reproductive age varied from US$ 1.0 in 2002 to US$ 6.3 in 2012. Expenditure on maternal and neonatal health per pregnant woman increased from US$ 34 in 2000 to US$ 512 in 2012, and per capita expenditure on under-five children increased from US$ 5.6 in 2000 to US$ 148.6 in 2012. Increased expenditure on RMNCH reflects a greater political support for RMNCH, along with greater emphasis on social assistance, family planning, and health reforms targeting poor areas, and a recent emphasis on antipoverty and crosscutting equitable policies and programmes focused on nutrition and maternal and neonatal mortality. Increasing domestic RMNCH expenditure likely enabled Peru to achieve substantial health gains. Peru can provide useful lessons to other countries struggling to achieve sustained gains in RMNCH by relying on their own health financing.

## Introduction

Investing in health at country level when accompanied by political will has been shown to result in significant progress not only in health outcomes, but also in individual and societal productivity and economic growth [1]. Moreover, such investment has the potential to improve health indicators in low-income and lower-middle-income countries to levels comparable to those presently seen in the best-performing middle-income countries [1]. This is particularly true for RMNCH improvements, especially where sustainable, relevant and equitable interventions are implemented at scale at country level [2].

Within the context of low- and middle-income countries, the magnitudes of official development assistance (ODA) investments in RMNCH at country level have been tracked and reported [3–5]. Studies on whether ODA in RMNCH was targeted to need have also been performed in several countries with different degrees of external aid dependence [4, 5].

However, to make progress in health outcomes, countries must not only rely on external funds, and their ability to leverage additional domestic funding will be critical. To date, neither there has been much less study of RMNCH expenditures at country level, nor an attempt to relate country level expenditures to outcomes [2, 6–8].

Similarly, there is little known about factors that might affect levels of domestic RMNCH expenditure [9]. These studies are important as they may reveal factors influencing resource allocation and expenditure, which may not be strictly related to need.

Peru is an upper-middle income country that has substantially reduced neonatal and under-five mortality (from 16.2 to 8 deaths per 1,000 livebirths and from 39.8 to 16.7 per 1,000 livebirths, respectively), as well as child stunting prevalence (from 31% to 17.5%) during the period 2000 to 2013 [10–12]. Within this context, a systematic analysis of changes in RMNCH financing overtime was largely overdue, to compare trends in financing levels to achievements in RMNCH outcomes. The economic, political, geographic and cultural context of Peru is explained in further detail elsewhere [10].

The Countdown to 2015 Peruvian RMNCH financing study was part of the Countdown to 2015 series of country case studies aimed at describing trends in RMNCH expenditure [10]. This paper examines trends in total health expenditure and out-of-pocket payments for health, domestic and external expenditure for RMNCH, assesses ratios between expenditure and coverage indicators, and explores posible drivers of RMNCH expenditure.

## Materials and methods

### Conceptual framework

**Fig 1:**
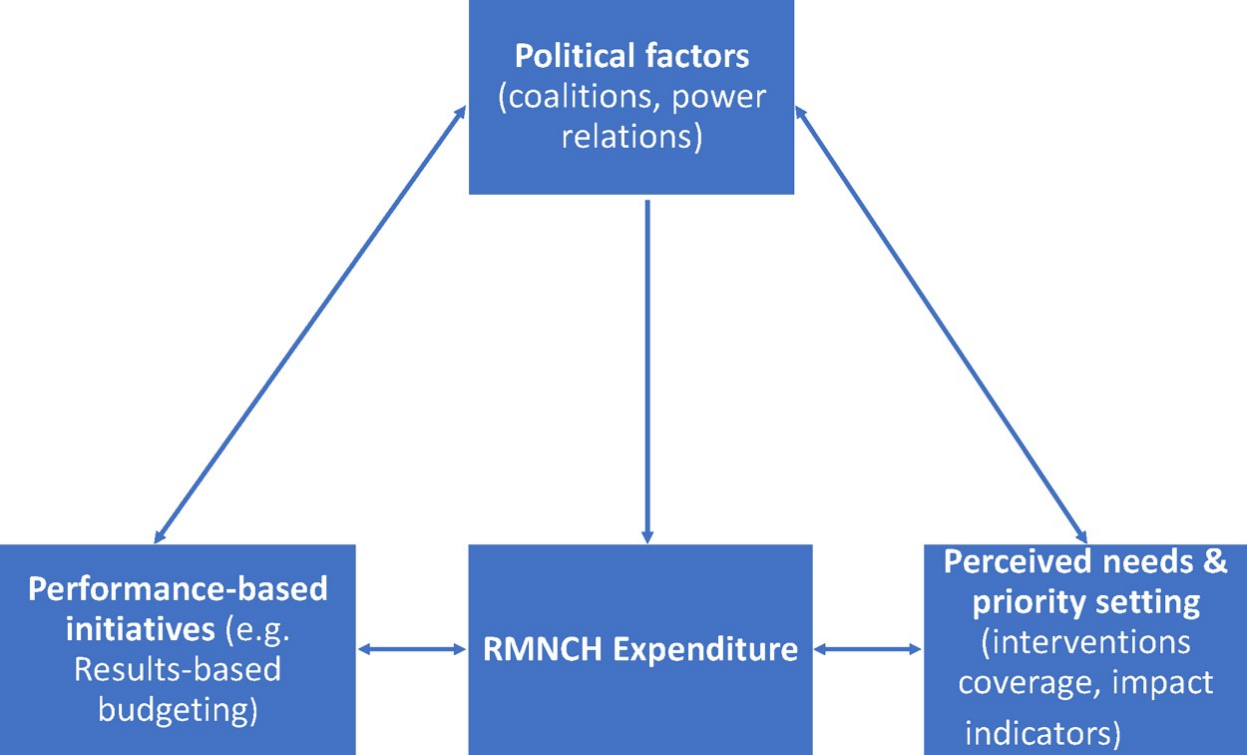
Conceptual framework for drivers of RMNCH expenditure.

We built a conceptual framework of RMNCH drivers through an iterative discussion process between members of the study team, which includes researchers familiar with health expenditure dynamics in Peru. This process was complemented by including insights from relevant literature on health financing drivers [13–17]. Our conceptual framework takes into account three main dimensions of drivers: political factors and policy-making processes, perceived needs and priorities, and performance–based initiatives in place (Fig 1). We elaborate on the relationship between these factors and RMNCH expenditure in the background theory section of the qualitative component of the study. The direction of the arrows in Fig 1 shows that actually there is not an exclusive one-way influence of a given set of factors on RMNCH expenditure, and that there is also a bidirectional influence between political and policymaking factors and other factors such as perceived needs and priorities and performance-based initiatives. The conceptual framework highlights the fact that political coalitions, power relations and advocacy are by far the most influential factors on RMNCH expenditure, and therefore a unidirectional arrow emphasizing such influence highlights this. We acknowledge that health financing occurs mainly through political agreements, and policy decisions are made by individuals positioned in government sectors such as the Ministry of Economy and Finance, who are not necessarily driven only by technical considerations such as the need to prioritize the health areas in most need. But on the other hand, Peru launched in 2007 a results-based budget initiative aimed at allocating funds to nationally established priority areas, namely maternal-neonatal health and child nutrition, on the basis of goals agreed with country regions in terms of progress in RMNCH interventions coverage and impact indicators such as neonatal and under-five mortality, and under-five stunting prevalence. Thus we felt that our conceptual framework should also incorporate this performance-based factor as possibly influential in the Peruvian RMNCH health financing process.

### Study setting

Peru has a two-tier health system comprising the public and private sectors. The public sector provides subsidized and semi-subsidized health services channeled by the Ministry of Health that is the main provider of primary, secondary and tertiary health care through the Comprehensive Health Insurance System (SIS) [18], and employment tax-based health services to the employees of the whole formal sector through the Health Social Security (Essalud) [19]. Those wihtout insurance who are poor receive free access to care [18]. The private sector includes not-for-profit and for-profit sub-sectors that provide health services that are not free at the point of care [19]. In 2000, the percentage of the population covered by either public or private insurance was only 24.4% [20]. By 2012, this percentage had increased to 61.8% (Essalud 24%, SIS 31.4%, and other insurance systems including private insurance 6.4%) [20]. Although Peru has made substantial progress in increasing access to health services at national and subnational levels, there are still substantial challenges to overcome in terms of coordination within the different subsystems, as well as in terms of decentralization, accountability, optimal financing, accessibility, equity and quality of care [21].

### Data sources

The study used a mixed methods approach. Quantitative data and methods were used to identify national time trends for expenditure on health, and the amount of RMNCH spending financed from ODA and from domestic spending. Various rounds of individual and group discussions were conducted to identify possible underlying factors influencing the variation of RMNCH expenditure over time.

### Quantitative methods

Multiple secondary data sources were utilized. To measure total health expenditure and government health expenditure we used an official National Health Accounts (NHA) report spanning the period 1995–2012 [22]. The out-of-pocket health spending (OOPS) and the total government health expenditure were obtained from the World Health Organization global health expenditure database for the years not available in the NHA report [23]. For RMNCH external funding, we used the Countdown database provided by Countdown to 2015 for the period 2003–2012 [3]. For domestic RMNCH expenditure data we used the information provided by Peru’s Ministry of Economy and Finance for the period 1999–2012 [24]. We were unable to measure OOPS for RMNCH due to the absence of a relevant data source. We ended up exploring different periods of time given constraints imposed by data availability from the different sources.

We calculated GDP per capita in constant 2012 US$ from the per capita figures in local currency (PEN) provided by the NHA report [22]. Data on coverage for RMNCH interventions was obtained from the Demographic and Health Surveys DHS [25].

For the expenditure series provided by the Ministry of Economy and Finance, expenditure data include a detailed description of activities relating to RMNCH. We classified expenditures into three groups: reproductive health, defined as expenditure on activities related to contraception, family planning, HIV and sexually transmitted diseases; maternal and newborn health, defined as expenditure on antenatal care, birth attendance and postnatal care activities; and child health defined as expenditure on preventative and curative activities targeted to under-five children excluding the neonatal period.

To classify RMNCH expenditures into these three categories, three independent reviewers reviewed and classified each activity provided by the Ministry of Economy and Finance. Discrepancies were resolved by consensus. As a result of the grouping process, the expenditure data periods were 2002–2012 for reproductive health, 2000–2012 for maternal-neonatal health, and 1999–2012 for child health. Per capita figures for expenditure on reproductive, maternal-neonatal and child health activities were obtained using as denominator the population of women of reproductive age, the population of pregnant women, and the under-five population, respectively [26, 27].

All per capita expenditures were estimated in constant 2012 US$ values to account for inflation. The Peruvian Central Bank deflators were used to convert the spending into 2012 soles, then the expenditures were converted into US$, by using the 2012 market exchange rates from the World Development Indicators database [28, 29].

### Qualitative methods

Factors underlying financial flows over time were assessed through several rounds of over 170 individual interviews and group discussions with experts from different sectors (national and regional governments, civil society, academia, and non-governmental organisations), particularly those familiar with RMNCH policy, programming and financing.

The background theory of the qualitative component is based on the conceptual framework developed to guide our analyses (Fig 1). In summary, it includes the assumptions described below.

1. RMNCH expenditure is influenced by domestic political factors and policy making processes. They comprise coalitions, power relations, advocacy, and how policy decisions on RMNCH expenditure are made. Different actors are considered, including the legislative, the executive, the regional and local governments, the media, the non-governmental organisations, and the community. We assumed these are the most influential factors driving RMNCH expenditure, even stronger than other technical factors such as the disease burden, or the perceived needs and priorities.
2. Perceived needs and priorities influence RMNCH expenditure. We assumed that RMNCH expenditures depend on the perceptions about the political priority RMNCH should receive and about the RMNCH burden (i.e. neonatal, infant and under-five mortality rates, and under-five stunting prevalence). We also assumed that low coverage levels in implementation of RMNCH interventions are perceived by policymakers as needs deserving particular strengthening. Perceived priorities also include internationally agreed priorities and initiatives highlighting maternal and child health as a global problem, which resonate at national level. Morevover, we assumed that the country economic profile in terms of GDP progress, poverty reduction policies, and access to basic facilities such as improved water and sanitation, also influence RMNCH expenditure.
3. Performance-based initiatives in place such as the results-based budgeting programmes influence RMNCH expenditure. They have been implemented in Peru since 2007, to stimulate a more efficient expenditure through effective implementation of evidence-based RMNCH interventions known to impact child survival and nutrition.
4. We furthermore assumed that underlying the remarkable reduction of neonatal and under-five mortality and of under-five stunting in Peru, there was a positive convergence of the above-mentioned factors to facilitate the implementation of effective policies, programmes and interventions directly or indirectly related to RMNCH, and that they also favoured a substantial increase in RMNCH expenditure, even if overall domestic health expenditure as a share of total government expenditure and of the GDP remained stagnant.

We used these assumptions to guide the interviews and the group discussions. They also served to prepare the main themes included in the interviews and group discussions, and as our analytical framework.

The interviews and group discussions were conducted before the quantitative analyses. To obtain the insight of individuals from different sectors, we organized two workshops in collaboration with the Ministry of Economy and Finance and The Pan American Health Organization, with the aim to discuss success factors that could explain the progress achieved by Peru in RMNCH, as well as the challenges faced. A special discussion section on drivers of RMNCH expenditure was included as part of the workshops, and as the main topic for the group discussions and interviews. Participants were formally invited through institutional letters.

Group discussions were set up jointly between organizers and participants during the workshops and were moderated by trained research team members. Individual interviews were conducted during and after the workshops by members of our research team. Both group discussions and interviews were conducted in ad-hoc places that provided privacy and comfort to participants. The researchers took notes of the group discussions and interviews, but these were not recorded.

### Analyses

Descriptive time trends were determined for total health expenditure, OOPS for health, external and domestic RMNCH funding. A linear regression was run to estimate the average annual rate of change of the variable of interest over the period. The percentage of change for the whole period was calculated by comparing the level in the end-year to that in the base-year.

To relate the magnitude of expenditure needed to obtain a unit of increase in coverage over time, we estimated ratios of domestic expenditure to coverage indicators by dividing per capita RMNCH expenditure by relevant coverage indicators. That is, we divided expenditure on reproductive health by family planning coverage, expenditure on maternal-neonatal health by antenatal and skilled birth attendance coverage, and child health expenditure by a composite coverage index (CCI). The CCI is a weighted mean of the coverage of eight preventive and curative interventions covering four steps of the continuum of care (family planning, maternity care, child immunization, and case management) that is described in more detail elsewhere [30]. To obtain the percentage of change in ratios over the study period, we estimated the cost per unit of coverage in the base-year, and the same for the end-year, and then expressed the percentage of change in the cost per unit of coverage.

The principal investigator (LH) and two other members of the study team categorized the information obtained through the interviews and group discussions into the themes established a priori on the basis of the background theory. If the messages did not fit into the pre-established categories, they were accomodated into new ones. A summary of findings was shared with participants through email and during further group meetings, for validation. Those that reached consensus between participants involved in the discussions were finally included by the study team as the underlying factors likely influencing financial flow.

A comprehensive desk review of factors possibly influencing health financial flow was additionally performed. For this, we asked all participants of the group discussions and interviews to provide information on unpublished documents and publications relevant to contextual factors and to RMNCH expenditure drivers from various government and non-government sectors (Ministry of Economy and Finance, Ministry of Health, Ministry of Development and Social Inclusion, PAHO, The World Bank, NGOs, and civil society organizations such as the Roundtable Against Poverty). Study team members collected, systematized and synthesized the information retrieved, to gain further understanding of the Peruvian context and of the national priorities and needs, so as to complement the insight obtained through the interviews and group discussions.

We broadly grouped financial flow driving factors emerging from the group and individual discussions into global and national factors, albeit they were most frequently interrelated. In summary, two periods could be clearly distinguished, with quite different political, policy-related and programmatic trends and approaches: pre-2000 and 2000 onwards.

### Ethical aspects

Our main quantitative analyses have been conducted on the basis of secondary data from the National Institute of Statistics and Computing, the DHS, the National Health Accounts, the Ministry of Economy and Finance, and the Countdown database provided by Countdown to 2015 for ODA spending. The datasets are aggregated, publicly available and are also anonymized whenever they originally involved individuals (DHS). We specify in the text of the paper each of the data sources utilized, as well as the corresponding website. All ethical issues were warranted by the institutions that originally led and conducted the surveys involving individual subjects (DHS).

Ethical review was not considered necessary for the qualitative component, since participants were being asked about administrative details in the course or as an extension of their work. No personal information was linked to the opinions expressed by participants to ensure their anonymity, and the results were grouped to avoid identification of individual participants.

## Results

### Trends in total health expenditure and expenditure by source

**Fig 2:**
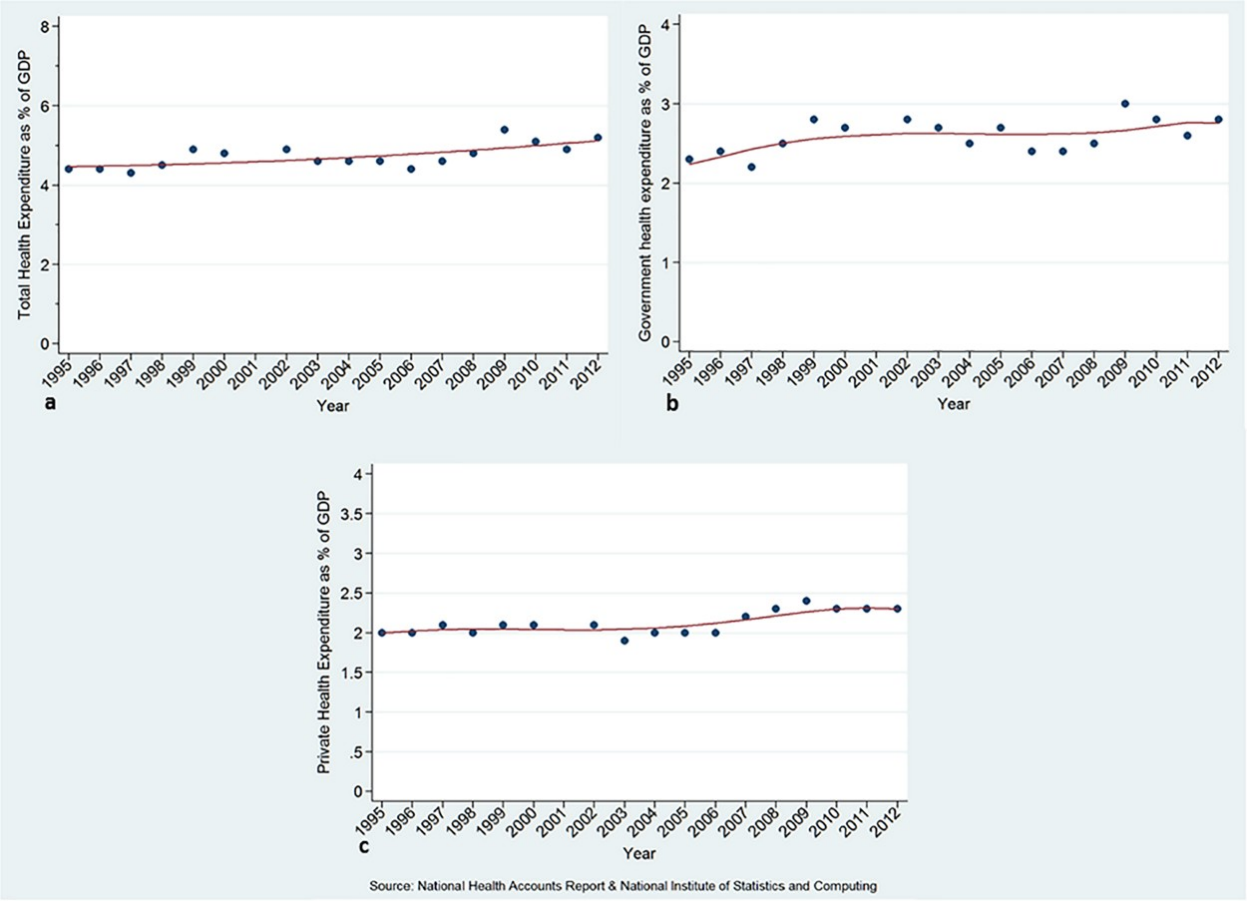
Total health expenditure (a), government health expenditure (b) and private health expenditure (c) as percentage of GDP.

Peru’s per capita total health expenditure (THE) remained a fairly constant share of GDP over this period from 4.4% in 1995 to 5.2% in 2012, and government health expenditure as a share of GDP varied from 2.3% to 2.8%, while private health expenditure remained at around 2% (Fig 2 and Table 1). Private health spending includes private health insurance, health spending by corporations (for-profit and not-for profit) and OOPS.

**Table 1:** Average annual rate of growth, and over entire period, health expenditure variables, Peru.

Expenditure indicator	Coefficient of annual variation		SE	p-value	Period	% change during the period
GDP per capita (constant 2012 US$)*	121.074		37.673	0.006	1995–2012	42.4
Total health expenditure as % of GDP	0.038		0.011	0.003	1995–2012	18.2
Per capita total health expenditure, (constant 2012 US$)	8.876		1.792	< 0.001	1995–2012	64.3
Government health expenditure as % of GDP	0.019		0.009	0.046	1995–2012	21.7
Government health expenditure as % of Total Government Expenditure	0.016		0.039	0.682	1995–2012	6.1
Per capita out-of-pocket spending, (constant 2012 US$)	2.811		0.782	0.002	1995–2012	51.8
Out-of-pocket spending as % of total health expenditure	0.000		0.121	0.998	1995–2012	-7.6
ODA expenditure per woman of reproductive age, (constant 2012 US$)	0.137		0.064	0.065	2003–2012	507.7
ODA expenditure per pregnant woman (constant 2012 US$)	0.144		0.473	0.768	2003–2012	-36.2
ODA expenditure per under-five child (constant 2012 US$)	0.217		0.546	0.701	2003–2012	21.9
Expenditure on reproductive health activities (constant 2012 US$/woman of reproductive age)	0.458		0.134	0.008	2002–2012	482.5
Expenditure on maternal-neonatal health activities (constant 2012 US$/pregnant woman)	30.308		4.262	< 0.001	2000–2012	1395.6
Expenditure on child health activities (constant 2012 US$/under-5 child)	10.314		1.882	< 0.001	1999–2012	966.7

*GDP used for comparison purposes. Values in columns 2, 3 and 4 come from the regressions for linear time trends.

**Fig 3:**
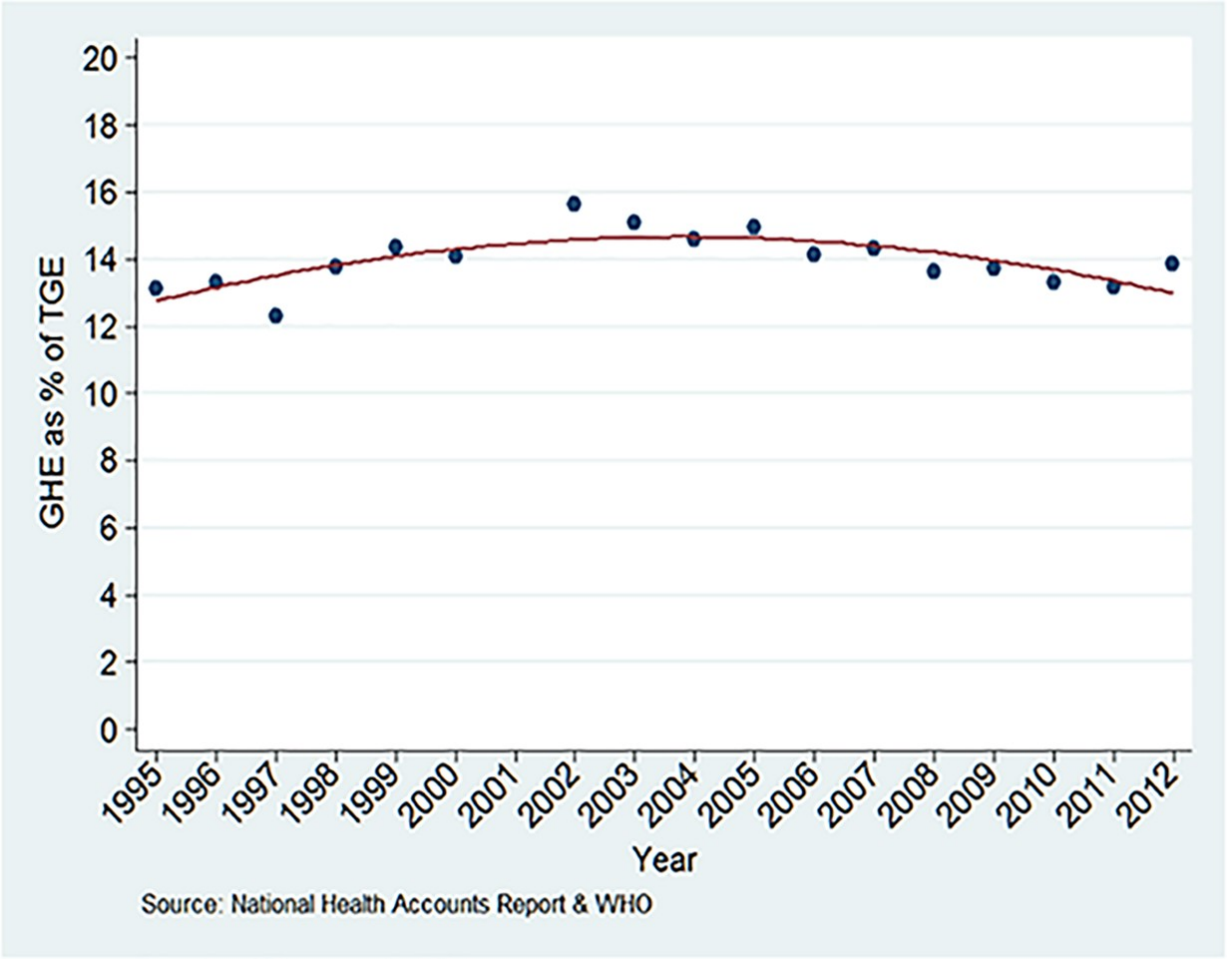
Government health expenditure (GHE) as percentage of total government expenditure (TGE).

**Fig 4:**
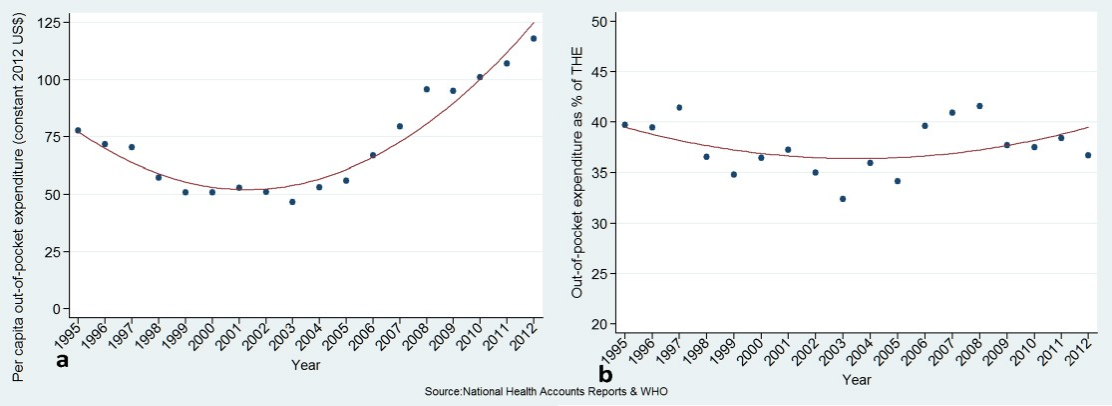
Out-of-pocket expenditure: per capita (a), and as percentage of total health expenditure (b).

Government health expenditure as a percentage of total government expenditure increased slightly from 13.1% in 1995 to 13.9% in 2012 (Fig 3 and Table 1). OOPS as a percentage of THE declined from 39.7% in 1995 to US$ 36.7% in 2012 (Fig 4 and Table 1), with a transient increase between years 2006 and 2008.

THE per capita had a higher relative increase over the study period than GDP per capita (64% versus 42%). Also, Government Health Expenditure as percentage of Total Government Expenditure increased slightly by 5.7% over time.

### Trends in external funding for RMNCH

**Fig 5:**
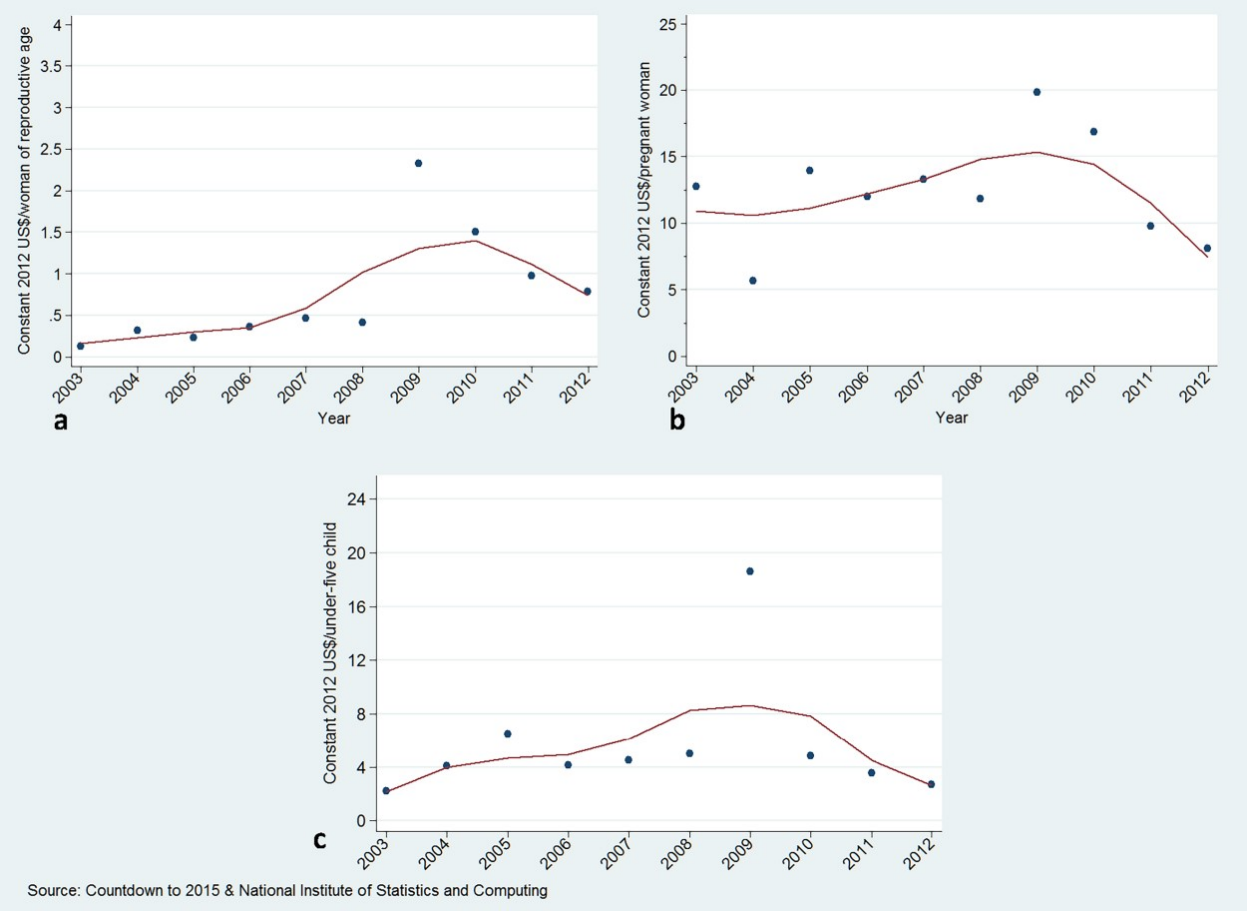
ODA expenditure on reproductive (a), maternal-neonatal (b) and child health (c).

ODA to reproductive and sexual health per woman of reproductive age increased from US$ 0.13 in 2003 to US$ 0.79 in 2012, while ODA to maternal and newborn health expenditure per pregnant woman decreased from US$ 12.7 in 2003 to US$ 8.1 in 2012, and ODA to child health per child under-five increased slightly from US$ 2.2 in 2003 to US$ 2.7 in 2012 (Fig 5 and Table 1).

**Fig 6:**
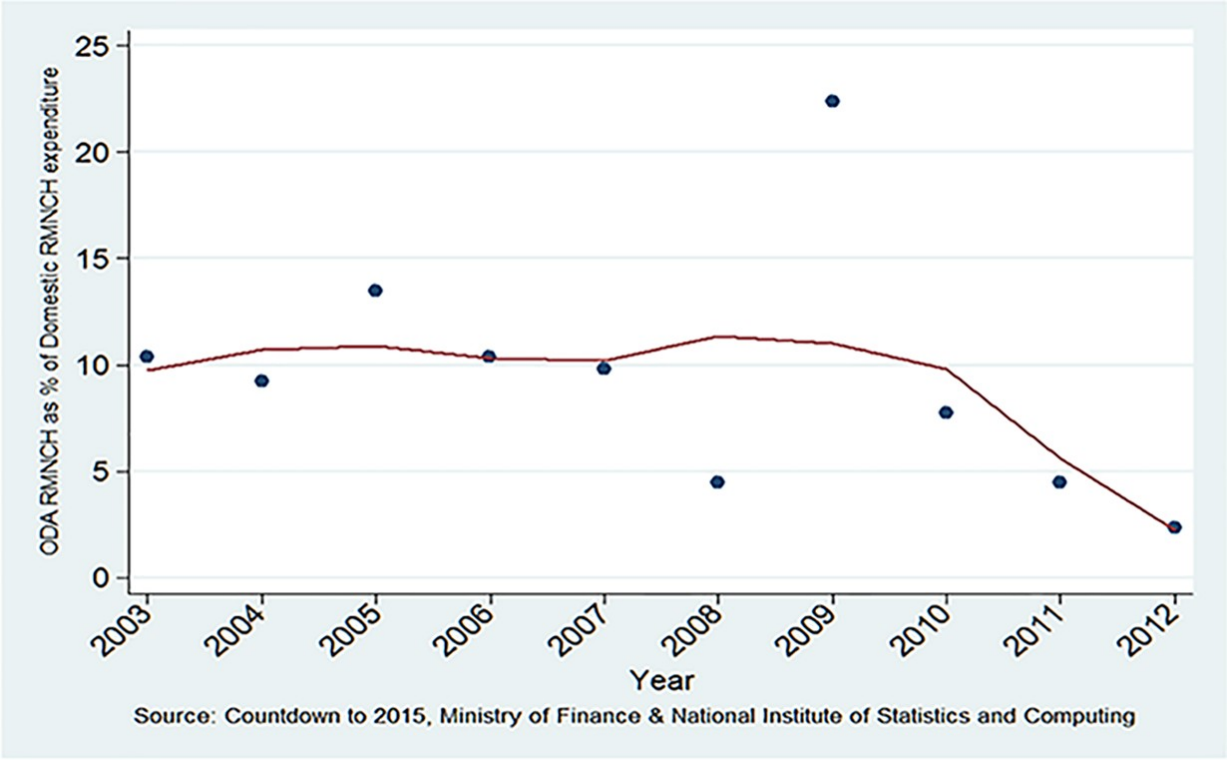
Total ODA expenditure on RMNCH as percentage of total domestic RMNCH expenditure.

Total ODA for RMNCH was 10.4% of total domestic RMNCH expenditure in 2003, and 2.4% in 2012, with some fluctuations in between (Fig 6 and Table 1).

The ODA disbursement to RMNCH increased by 17% over the study period. The percentage of increase for reproductive health over the study period was 498%. Corresponding ODA changes for maternal-neonatal health and for child health were -36% and 22%, respectively.

### Trends in domestic RMNCH expenditure over time

**Fig 7:**
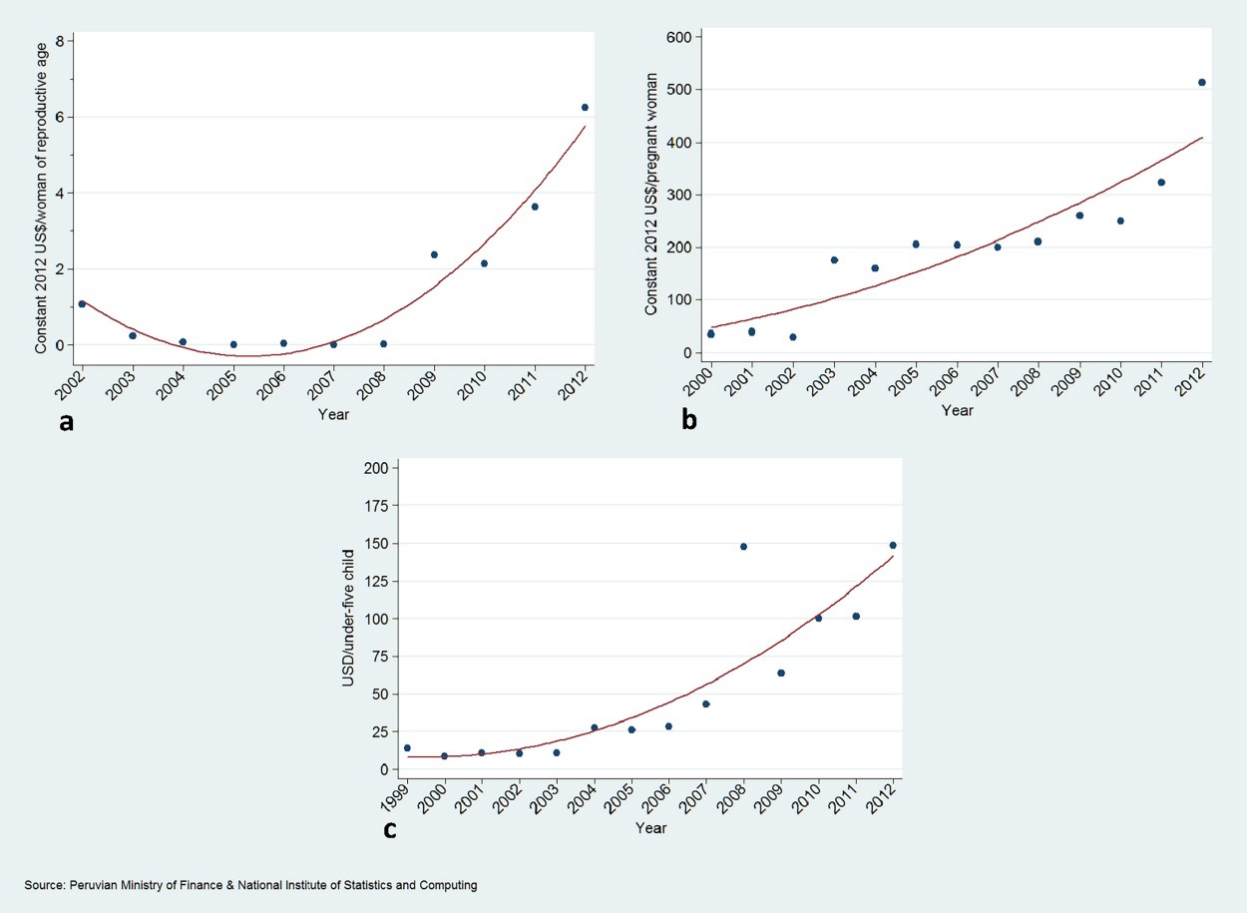
Domestic expenditure on reproductive health (a), maternal-neonatal health (b) and child health (c).

Fig 7, Table 1 show time trends in per capita domestic expenditure on reproductive, maternal-neonatal and child health in constant 2012 US$.

Domestic reproductive health expenditure per woman of reproductive age increased by about six times from US$ 1.0 in 2002 to US$ 6.3 in 2012, with a transient decline from 2002 to 2006.

Domestic maternal and newborn health expenditure per pregnant woman increased more than 15 times from US$ 34.0 in 2000 to US$ 512.0 in 2012. The largest growth in expenditure was for child health per child under-five which increased more than 17 times from US$ 5.6 in 2000 to US$ 148.6 in 2012. All trends were visually steeper from 2008 onwards.

### Changes in ratios of domestic expenditure to coverage indicators

Table 2 shows the annual ratios of specific RMNCH domestic expenditure to relevant coverage indicators over the study periods.

**Table 2:** Change of ratio of domestic RMNCH expenditure to RMNCH coverage, Peru.

RMNCH expenditure indicator and coverage indicator		Change of expenditure/coverage ratio over time (%)	Period
**Expenditure per woman of reproductive age** **(constant 2012 US$)**			
	% of women with family planning satisfied	9382.2	2004–2012
**Expenditure per pregnant woman** **(constant 2012 US$)**			
	% of women with at least 4 antenatal care visits	985.2	2000–2012
	% of live births attended by skilled birth attendants	922.9	2000–2012
**Expenditure per under-five child** **(constant 2012 US$)**			
	Composite coverage index	1500.4	2000–2012

The ratio of reproductive health expenditure per woman of reproductive age to family planning coverage increased by more than 94 times between 2004 and 2012. The ratio of expenditure on maternal-neonatal health per pregnant woman to antenatal care coverage increased by about 11 times. The ratio of expenditure on maternal-neonatal health per pregnant woman to skilled birth attendance increased by more than 10 times. The ratio of expenditure per child health to CCI increased by 16 times.

### Qualitative results

There was consensus among participants that increased expenditure on RMNCH reflects a greater political support for RMNCH, along with an increased emphasis on social assistance, family planning, and health reforms targeting poor areas, and a more recent emphasis on antipoverty and crosscutting equitable policies and programmes targeting nutrition and maternal and neonatal mortality.

According to the interviews and group discussions, corroborated by the literature review, during the period prior to 2000s, social assistance and fertility reduction policies were widely promoted by international organizations [31–33], which were translated at national level in the implementation of food assistance and family planning programmes [34, 35]. With regard to child health, vertical programmes were globally promoted [36–38], and were followed by country level financing and implementation of growth monitoring, immunization, diarrhoeal disease prevention, and acute respiratory infections programmes.

Participants emphasized that during the post-2000 period, global level focus on poverty reduction and on health reforms to reach poor segments of the population [39] ran parallel to national level focus on financing of anti-poverty initiatives such as the conditional cash transfer JUNTOS [40], and of comprehensive health insurance systems such as Seguro Integral de Salud (SIS) [18]. While in terms of RMNCH, global emphasis on poverty reduction and health reforms may have acted as driving forces for higher investment in integrated strategies such as the Integrated Management of Childhood Illness aimed at reducing stunting and child mortality [41].

According to participants, more recently strong international impetus was given to results-based budgeting, to increase the efficiency and effectiveness of expenditure in the public sector [42]. Likewise, participants concurred that a strong civil society advocacy for equitable evidence-based interventions focused on child nutrition, mothers and newborns [43–45], led in Peru to sustained investment in crosscutting comprehensive programmes inspired in this approach. They were implemented from 2007 onwards, including the Strategic Maternal-Neonatal Programme aimed at improving maternal and neonatal health [46,47], and the Articulate Nutritional Programme seeking to ensure the sustainability of stunting reduction interventions [48], and to increase the efficiency of the RMNCH expenditure at national and regional level.

## Discussion

The evolution of domestic health expenditure over the last two decades in Peru shows that it progressively relied more on its own funds than on donor aid, which is a remarkable achievement.

Interestingly, Peru mobilized significant domestic funds directed particularly to RMNCH, OOPS remained high and health spending as a share of GDP remained constant. This might be reflecting that access to affordable and quality health care needs still further improvement, to ensure sustainability of the progress achieved by Peru in RMNCH.

Domestic expenditure on RMNCH increased substantially in Peru during the study period, with particularly steep progress from 2008 onwards, reflecting a greater priority given to RMNCH by politicians at the central level. This remarkable increase, which was more dramatic for maternal-neonatal and child health, has likely contributed to the impressive reduction in under-five and neonatal mortality, and in child stunting, which occurred in Peru in the last few decades. Of note, while there was an increase in reproductive health expenditure, it was less pronounced than that observed in maternal, neonatal and child health, which might reflect concerns about violation of human rights during the implementation of family planning programmes during the 1990s [49], and as a consequence, a lower visibility of reproductive and sexual health during the early 2000s ]and afterwards [34].

The ratios of expenditure to coverage indicators showed that the amount of money spent to obtain a unit of increase in coverage increased over time. This does not necessarily mean lower efficiency, but reveals more likely that the higher the progress reached in intervention coverage, the higher the amount of spending increase needed to obtain a comparable or even lower pace of progress.

The synthesis of the qualitative and desk review information obtained might suggest that an increase of total health and RMNCH expenditure was driven by specific underlying factors discussed below, with progressive focus on the poorest segments of the population.

During the 1990s, the global aim of fertility reduction and improvement of food security seemed to result in increased government financial investment to implement at scale family planning and food supplementation programmes focused on the poorest rural areas [34, 35]. During the early 2000s, family planning interventions had lower political visibility, and higher priority was given to the reduction of under-five mortality through integrated strategies such as the Integrated Management of Childhood Illnesses [41], and more recently to the reduction of under-five stunting and to improvement in maternal and neonatal health, which was in line with global evidence advocating the implementation of evidence-based comprehensive RMNCH interventions.

Important driving forces that influenced an increased financial investment in RMNCH health during the 2000s, included the adoption of national and sub-national political commitments pushed by a strong civil societal advocacy, which placed RMNCH at top of the political agenda, needing crosscutting and multidisciplinary action [43–45] This was translated into the implementation of sustained anti-poverty and social inclusion policies and programmes, in the commitment to specific RMNCH goals, and in the implementation of specific RMNCH interventions [46–48].

We wonder whether the increase of financial resources over time may have influenced the quality of the RMNCH interventions, which is one of the objectives of the crosscutting maternal-neonatal and nutritional programmes led by the Ministry of Economy and Finance [47,48]. Unfortunately, the DHS on which our study is partly based do not provide information about the quality of the RMNCH interventions.

Our study clearly shows that Peru relied basically on its domestic expenditure for the progress achieved in RMNCH, with ODA as a minor component of the financial support. This is in contrast with other Countdown countries, which rely extensively on external support [50]. We must point out however that Peru is one of very few upper-middle income Countdown countries and therefore was more able to achieve this. It cannot be overemphasized that ensuring sustainability of progress will require that countries resort in the long-term to domestic financing, after the necessary initial external aid.

Peru compares favorably with several other Countdown countries in terms of a positive trend of per capita total health expenditure, which increased dramatically from Int$ 207 in 1995 to Int$ 555 in 2012 [50], and a substantial proportion of this expenditure was accounted for by RMNCH financing.

However, while the Peruvian experience on financial support of RMNCH is to be praised as a positive story that offers important lessons to other countries, some important caveats need also to be addressed, as they may hamper further progress in the future. First, total and government health expenditure as percentage of GDP remained almost without change at low levels, while government health expenditure as percentage of total government expenditure declined from 17% to less than 14%. Although the increase in specific RMNCH expenditure and the progress in RMNCH coverage and impact indicators indicate higher priority given to RMNCH, there is the need to further discuss the relevance of increasing the overall budget of the public health sector as the main provider of health services in Peru, including those related to reproductive, maternal, neonatal and child health. Second, OOPS as percentage of total health expenditure is still too high, about 37% in 2012, reflecting very likely remaining constraints in access, utilization and quality of health care. A substantial reduction of OOPS will be necessary in a near future to reduce the risk of catastrophic spending, which may otherwise hamper the country efforts to further reduce poverty.

We must acknowledge some limitations of our study. First, we used different data sources with different time periods and differing consistency, which need to be addressed,to ensure more robust analyses in the future. Second, we could not obtain data on OOPS for RMNCH, which could have illuminated the specific burden of potentially catastrophic spending when families face problems related to reproductive, maternal, neonatal and child health. Finally, there were no data available on quality of RMNCH interventions such as antenatal care visits, although the Peruvian DHS has started to obtain them recently, allowing future assessment of associations between RMNCH spending and quality of care.

In conclusion, Peru is an illustrative example of progressively higher emphasis given to domestic health expenditure and to RMNCH expenditure in particular, and can provide useful lessons to other countries struggling to achieve sustained gains in maternal and child health by relying on their own financial flows.

## References

[1] Jamison DT, Summers LH, Alleyne G, Arrow KJ, Berkley S, Binagwaho A, et al Global health 2035: a world converging within a generation. Lancet. 2013;382(9908):1898–955. 10.1016/S0140-6736(13)62105-4 24309475

[2] Stenberg K, Axelson H, Sheehan P, Anderson I, Gulmezoglu AM, Temmerman M, et al Advancing social and economic development by investing in women's and children's health: a new Global Investment Framework. Lancet. 2014;383(9925):1333–54. 10.1016/S0140-6736(13)62231-X 24263249

[3] Arregoces L, Daly F, Pitt C, Hsu J, Martinez-Alvarez M, Greco G, et al Countdown to 2015: changes in official development assistance to reproductive, maternal, newborn, and child health, and assessment of progress between 2003 and 2012. Lancet Glob Health. 2015;3(7):e410–21. 10.1016/S2214-109X(15)00057-1 26087987

[4] Hsu J, Berman P, Mills A. Reproductive health priorities: evidence from a resource tracking analysis of official development assistance in 2009 and 2010. Lancet. 2013;381(9879):1772–82. 10.1016/S0140-6736(13)60762-X 23683644

[5] Hsu J, Pitt C, Greco G, Berman P, Mills A. Countdown to 2015: changes in official development assistance to maternal, newborn, and child health in 2009–10, and assessment of progress since 2003. Lancet. 2012;380(9848):1157–68. 10.1016/S0140-6736(12)61415-9 23000291

[6] Marten R, McIntyre D, Travassos C, Shishkin S, Longde W, Reddy S, et al An assessment of progress towards universal health coverage in Brazil, Russia, India, China, and South Africa (BRICS). Lancet. 2014;384(9960):2164–71. 10.1016/S0140-6736(14)60075-1 24793339 PMC7134989

[7] The Partnership for Maternal NaCH. The PMNCH 2014 Accountability Report. Tracking Financial Commitments to the Global Strategy for Women’s and Children’s Health. [cited 2015 August 08]. Available from: http://www.who.int/pmnch/knowledge/publications/pmnch_report14.pdf?ua=1.

[8] The Partnership for Maternal Newborn and Child Health. The PMNCH 2014 Accountability Report. Tracking Financial Commitments to the Global Strategy for Women’s and Children’s Health. 2014 [cited 2015 August 08]. Available from: http://www.who.int/pmnch/knowledge/publications/pmnch_report14.pdf?ua=1.

[9] Lu C, Schneider MT, Gubbins P, Leach-Kemon K, Jamison D, Murray CJ. Public financing of health in developing countries: a cross-national systematic analysis. Lancet. 2010;375(9723):1375–87. 10.1016/S0140-6736(10)60233-4 20381856

[10] Huicho L, Segura ER, Huayanay-Espinoza CA, de Guzman JN, Restrepo-Mendez MC, Tam Y, et al Child health and nutrition in Peru within an antipoverty political agenda: a Countdown to 2015 country case study. The Lancet Global health. 2016;4(6):e414–26. 10.1016/S2214-109X(16)00085-1 27198845

[11] Huicho L, Huayanay-Espinoza CA, Herrera-Perez E, Nino de Guzman J, Rivera-Ch M, Restrepo-Mendez MC, et al Examining national and district-level trends in neonatal health in Peru through an equity lens: a success story driven by political will and societal advocacy. BMC public health. 2016;16 Suppl 2:796. 27634453 10.1186/s12889-016-3405-2 PMC5025833

[12] Huicho L, Huayanay-Espinoza CA, Herrera-Perez E, Segura ER, Nino de Guzman J, Rivera-Ch M, et al Factors behind the success story of under-five stunting in Peru: a district ecological multilevel analysis. BMC Pediatr. 2017;17(1):29 10.1186/s12887-017-0790-3 28103825 PMC5248498

[13] Unicef. Analysis of the political economy of health, particularly reproductive, maternal, newborn and child health, in four countries of South and East Asia. Available from: https://www.unicef.org/videoaudio/PDFs/UNICEF_Working_Paper_on_political_economy_analysis_in_the_health_sector_-_27Aug15.pdf.

[14] The World Health Organization. Health Service Planning and Policy-Making. A toolkit for Nursed and Midwives. Module 4. Policy-Development Process [Available from: http://www.wpro.who.int/publications/docs/hsp_mod4_1E08.pdf.

[15] Leider JP, Tung G, Castrucci B, Sprague JB. Health spending and political influence: the case of earmarks and health care facilities. J Public Health Manag Pract. 2015 Mar-Apr;21(2):161–6. 10.1097/PHH.0000000000000116 25148133 PMC4336334

[16] Malik MA, Nahyoun AS, Rizvi A, Bhatti ZA, Bhutta ZA. Expenditure tracking and review of reproductive maternal, newborn and child health policy in Pakistan. Health Policy Plan. 2017 7 1;32(6):781–790. 10.1093/heapol/czx021 28334970

[17] Mann C, Ng C, Akseer N, Bhutta ZA, Borghi J, Colbourn T, Hernández-Peña P, Huicho L, Malik MA, Martinez-Alvarez M, Munthali S, Salehi AS, Tadesse M, Yassin M, Berman P; Countdown to 2015 Health Finance Working Group. Countdown to 2015 country case studies: what can analysis of national health financing contribute to understanding MDG 4 and 5 progress?. BMC Public Health. 2016 9 12;16 Suppl 2:792 10.1186/s12889-016-3403-4 27634209 PMC5025819

[18] Ministerio de Salud del Perú. Seguro Integral de Salud. SIS. [Available from: http://www.sis.gob.pe/portal/index.html.

[19] Alvarado B, Mrazek, M. Health Outcomes and Public Health Sector Performance. In: Giugale MM, Fretes-Cibils V, Newman, JL. An Opportunity for a Different Peru.Prosperous, Equitable, and Governable. 2007 [Available from: https://openknowledge.worldbank.org/bitstream/handle/10986/6633/382860ENGLISH0101OFFICIAL0USE0ONLY1.pdf?sequence=1&isAllowed=y.

[20] Ministerio de Salud del Perú y Consorcio de Investigación Económica y Social. Cuentas Nacionales de Salud Perú, 1995–2005. [Available from: http://www.who.int/nha/country/per/NHA_Peru_1995-2005.pdf.

[21] Polastry R, Rojas, F. Decentralization. In: Cotlear, D. A New Social Contract for Peru. An Agenda for Improving Education, Health Care, and the Social Safety Net. 2007 [Available from: http://siteresources.worldbank.org/INTPCENG/Resources/A_New_Social_Contract_for_Peru.pdf.

[22] Ministerio de Salud. Cuentas Nacionales en Salud, Perú 1995–2012 2015 [Available from: http://bvs.minsa.gob.pe/local/MINSA/3248.pdf.

[23] World Health Organization. Global Health Expenditure Database [Available from: http://apps.who.int/nha/database/Select/Indicators/en.

[24] Ministerio de Economía y Finanzas. Sistema de Administración Financiera (SIAF) [Available from: http://apps5.mineco.gob.pe/transparencia/Navegador/default.aspx.

[25] Instituto Nacional de Estadistica e Informatica. Microdatos. Base de Datos. [Available from: http://iinei.inei.gob.pe/microdatos/.

[26] Instituto Nacional de Estadística e Informática. Perú: Estimaciones y Proyecciones de Población Urbana y Rural por Sexo y Edad Quinquenales, según Departamento, 2000–2015 2009 [Available from: http://proyectos.inei.gob.pe/web/biblioineipub/bancopub/Est/Lib0844/index.htm.

[27] Instituto Nacional de Estadistica e Informatica. Series Nacionales. Población Estimada y Proyectada. Población Total [Available from: http://series.inei.gob.pe:8080/sirtod-series/.

[28] The World Bank. World Development Indicators [Available from: http://data.worldbank.org/data-catalog/world-development-indicators.

[29] The World Bank. World Development Indicators 2015 [Available from: http://databank.worldbank.org/data/reports.aspx?source=world-development-indicators-s_g.

[30] Barros AJ, Victora CG. Measuring coverage in MNCH: determining and interpreting inequalities in coverage of maternal, newborn, and child health interventions. PLoS medicine. 2013;10(5):e1001390 10.1371/journal.pmed.1001390 23667332 PMC3646214

[31] The World Bank. The Global Family Planning Revolution. Three Decades of Population Policies and Programs 2007 [Available from: http://siteresources.worldbank.org/INTPRH/Resources/GlobalFamilyPlanningRevolution.pdf.

[32] Cleland J, Berstein S, Ezeh A, Faundes A, Glasier A, Innis J. Family planning: the unfinished agenda. The Lancet. 2006;368(9549):1810–27. 10.1016/S0140-6736(06)69480-4 17113431

[33] The World Bank. Poverty and hunger: issues and options for food security in developing countries. 1986 [Available from: http://www-wds.worldbank.org/external/default/WDSContentServer/WDSP/IB/1999/09/17/000178830_98101901455676/Rendered/PDF/multi_page.pdf.

[34] Gribble JN, Sharma S, Menotti EP. Family planning policies and their impacts on the poor: Peru's experience. Int Fam Plan Perspect. 2007;33(4):176–81. 10.1363/ifpp.33.176.07 18178542

[35] Valdivia M. Peru: is identifying the poor the main problem in targeting nutritional programs? 2005 [Available from: http://siteresources.worldbank.org/HEALTHNUTRITIONANDPOPULATION/Resources/281627-1095698140167/RPP7ValdiviaPeruFinal.pdf.

[36] Claeson M, Waldman RJ. The evolution of child health programmes in developing countries: from targeting diseases to targeting people. Bull World Health Organ. 2000;78(10):1234–45. 11100618 PMC2560618

[37] The World Bank. World Development Report: Investing in Health 1993 [Available from: https://openknowledge.worldbank.org/handle/10986/5976.

[38] The World Bank. World Development Report 2000/2001: Attacking Poverty [Available from: https://openknowledge.worldbank.org/handle/10986/11856.

[39] Victora CG, Wagstaff A, Schellenberg JA, Gwatkin D, Claeson M, Habicht JP. Applying an equity lens to child health and mortality: more of the same is not enough. Lancet. 2003;362(9379):233–41. 10.1016/S0140-6736(03)13917-7 12885488

[40] Ministerio de Desarrollo e Inclusión Social (MIDIS). Programa Nacional de Apoyo Directo a los más Pobres. [Available from: http://www.juntos.gob.pe/.

[41] Ministerio de Salud. Los diez primeros años de AIEPI en el Perú 2006 [Available from: http://www.bvsde.paho.org/texcom/AIEPI/AIEPI10Peru.pdf.

[42] The World Bank. Results-Based Budgeting: Achieving Effective and Efficient Public Service 2010 [Available from: http://siteresources.worldbank.org/NEWS/Resources/FormatResults2010-PREM-SB-New-Results-BasedBudgeting.pdf.

[43] Acuerdo Nacional. Unidos Para Crecer [Available from: http://acuerdonacional.pe/.

[44] Mesa de Concertación de Lucha Contra la Pobreza. Acuerdos de Gobernabilidad. El Cumplimiento de la Palabra. 2006 [Available from: http://elecciones.mesadeconcertacion.org.pe/static/download/Acuerdos_de_Gobernabilidad_el_cumplimiento_de_la_palabra_ano_2006.pdf.

[45] Mesa de Concertación de Lucha Contra la Pobreza. Seguimiento Concertado a los Acuerdos de Gobernabilidad. Guía Metodológica para el Seguimiento a la Ejecución del Presupuesto Público 2006 [Available from: http://www2.mesadeconcertacion.org.pe/static/upload/file/publicacion_d2594580fd5bbd112e3432dd2209b811.pdf.

[46] Mesa de Concertación de Lucha Contra la Pobreza. Programa Estratégico "Salud Materno Neonatal". Reporte de seguimiento concertado: Evaluación del año 2009. [Available from: http://www.mesadeconcertacion.org.pe/documentos/documentos/doc_01465.pdf.

[47] Mesa de Concertación de Lucha Contra la Pobreza. Programa Presupuestal Salud Materno Neonatal.http://www.mesadeconcertacion.org.pe/documentos/documentos/doc_01695.pdf

[48] Ministerio de Economía y Finanzas. Programa Articulado Nutricional 2007 [Available from: http://www.mef.gob.pe/index.php?option=com_content&view=article&id=2144:salud-materno-neonatal&catid=211&Itemid=101528.

[49] Miranda J. Reproductive health without rights in Peru. The Lancet. 2004;363(9402):68–9. 10.1016/S0140-6736(03)15175-6 14724000

[50] Countdown to 2015. Fulfilling the health agenda. The 2014 Report. [Available from: http://www.countdown2015mnch.org/documents/2014Report/Countdown_to_2015-FulfillingtheHealth_Agenda_for_Women_and_Children-The_2014_Report-Conference_Draft.pdf. 10.1016/S0140-6736(14)60925-9 PMC7613194 24990815

